# JBO 2025 List of Reviewers

**DOI:** 10.1117/1.JBO.31.1.010101

**Published:** 2026-01-29

## Abstract

Thanks to reviewers who served JBO in 2025.

The *Journal of Biomedical Optics* sincerely thanks the following individuals who served as reviewers in 2025. The success of our publication hinges on the voluntary contributions of time and energy put forth by these professionals.

Ola Abdalsalam
Robert Adamson
Steven Adie
Salavat Aglyamov
Vasundhara Agrawal
Walter Akers
Ata Akin
Dai Akita
Aneesh Alex
Yogeshwari Ambekar
Stefan Andersson-Engels
Rinat Ankri
Giuseppe Antonacci
Lorilee Arakaki
Hamidreza Asemani
Xavier Attendu
Maximilian Aumiller
Kamran Avanaki
Wesley Baker
Ganesh Balasubramaniam
Wagner Balthazar
Timothy Baran
Luca Baratelli
Jennifer Barton
Mikhail Berezin
Renzhe Bi
Protik Chandra Biswas
Brecken Blackburn
Richard Blackmon
Giles Blaney
George Blassan
Richard Bouchard
Audrey Bowden
Stephen G. Bown
Denis Bragin
Jonathan Brand
David Brenes
Juan Briñez
J. Quincy Brown
David Busch
Alexander Bykov
Andreea Campu
Honghao Cao
Jiaming Cao
Xu Cao
Stefan Carp
Dmitrijs Celinskis
Jonathan Celli
Jaepyeong Cha
Shuang Chang
Defu Chen
Fang Chen
Lei Chen
Nanguang Chen
Qiang Chen
Sung-Liang Chen
Yanyan Chen
Yu Chen
Yuxuan Cheng
Ata Chizari
Bernard Choi
Hak Soo Choi
Seongwook Choi
Elise Colin
Tim Collins
Lorenzo Cortese
Bruna Cotrufo
Joao Couto
Ana Maria Craciun
Christian Crouzet
Han Cui
Cuixia Dai
Michael Daly
Laurence Danis
Rupsa Datta
Johannes de Boer
Edward Delikatny
Valentin Demidov
Handi Deng
Qilin Deng
Mamadou Diop
Joris Dirckx
Alexander Doronin
Alexandre Douplik
Tanja Dragojevic
Virgil-Florin Duma
Andrey Dunaev
Cody Dunn
Nicholas Durr
Kareem Elsayad
Ismail Erbas
Olga Eremina
Rinat Esenaliev
Muaaz Faiyazuddin
Qiyin Fang
Inas Faris
Joyce Farrell
Jinchao Feng
Donald Fernandes
Jane Frederick
Ingemar Fredriksson
Daniel Fried
Lei Fu
Thomas Gallot
Fei Gao
Feng Gao
Xin Gao
Héctor García
Manas Gartia
Emily Gawrys
Jean-Luc Gennisson
Irene Georgakoudi
Michael Giacomelli
Dimitris Gorpas
Valeria Grasso
Éléa Gros
Viktor Gruev
Ireneusz Grulkowski
Shravan Gupta
Eugenio Gutierrez
Jose Alberto Gutiérrez
Lina Hacker
Robert Hadfield
Ghassan Hamarneh
Niko Hanninen
Jahid Hassan
Maryam Hatami
Carole Hayakawa
Feng He
Honghui He
Jingyi He
Ahmed Heikal
Bradley Henderson
Joshua Herzog
Xiaoming Hu
Yong Huang
Kristie Huda
Thomas Hutchens
Leanne Iannucci
Masatoshi Ichikawa
Nicusor Iftimia
Hubert Jean-Ruel
Hongbo Jia
Mengyu Jia
Jingjing Jiang
Shan Jiang
Johannes Johansson
Francis Kalloor Joseph
Amit Joshi
Irina Kabakova
Dongkyun Kang
Dariush Kari
Kavon Karrobi
Giulia Kassab
Md Rejvi Kaysir
Gordon Kennedy
Alexander Khmaladze
Geon Kim
Jeesu Kim
Mikhail Kirillin
Andrew Kirk
Ivan Kosik
Nikola Krstajic
Carla Kulscar
Shashikant Lahade
Xiaomin Lai
Gabrielle Laloy-Borgna
Jesse Lam
Frederic Lange
Maryse Lapierre-Landry
Kirill Larin
V. N. Du Le
Changho Lee
Hyeon Jeong Lee
Min Young Lee
Terence Leung
Buhong Li
Changhui Li
Guoqiang Li
Guoyang Li
Haoyu Li
Jiao Li
Lei Li
Shuying Li
Ting Li
Xingde Li
Yingguang Li
Zhe Li
Lothar Lilge
Li-En Lin
Weichiang Lin
Chao Liu
Gangjun Liu
Hanli Liu
Jun Liu
Li Liu
Wenhui Liu
Xuan Liu
Yudi Liu
Yurong Liu
Zhongyu Liu
Angel Lizana
Andrea Locke
Geoffrey Luke
Gang Luo
Ling Ma
Suodong Ma
Megan Madonna
Steen Madsen
Marcello Magri Amaral
Simon Mahler
Nataliya Makeeva
Srivalleesha Mallidi
Rafal Mantiuk
Rayyan Manwar
Agathe Marmin
Edward Maytin
Robert McLaughlin
Laurie McNeil
Igor Meglinski
Kristen Meiburger
Ratheesh Kumar Meleppat
Luca Mennozi
Peng Miao
Daniel Milej
David Miller
Shahed Mohammed
Guillermo Monroy
Claudia Mota
Mircea Mujat
Marie Muller
Anupama Nair
Kohei Nakajima
Sreyankar Nandy
Hieu Nguyen
John Quan Nguyen
Thien Nguyen
Shuibin Ni
Mark Niedre
Line Nielsen
Navid Ibtehaj Nizam
Farouk Nouizi
Marien Ochoa Mendoza
Paul Okagbare
Elizabeth Olson
Bernhard Ortel
Frederic Pain
Rahul Pal
Uttam Pal
Daisong Pan
Rishikesh Pandey
Sanjana Pannem
Eirini Papagiakoumou
Arturo Pardo
Evgueni Parilov
YongKeun Park
Asha Parmar
Ashwin Parthasarathy
Nishigandha Patil
Ivan Pelivanov
Xiaorui Peng
Vijitha Periyasamy
T. Joshua Pfefer
Thi-Thu-Hien Pham
Thinh Pham
Thinh Phan
Qi Pian
Daqing Piao
Mark Pierce
Yu Ping
Michael Pircher
Brian Pogue
Monica Potara
Jaya Prakash
Buky Wahyu Pratama
Robert Prevedel
Ruobing Qian
Zhen Qiu
Junle Qu
Timothy Quang
Zachery Quince
Matin Raayai Ardakani
Uma Maheswari Rajagopalan
Praveenbalaji Rajendran
Jessica Ramella-Roman
Evelio E. Ramirez-Miquet
Sweta Rani
Divya Ravi
Siddharth Rawat
Rebecca Re
Karen Reiser
Wuwei Ren
Tom Rix
Constance Robbins
Dominic Robinson
Mitchell Robinson
Darren Roblyer
Andres Rodriguez
Omar Rodriguez-Nunez
Katy Roodenko
Daan Roos
Eben Rosenthal
E. Victor Ross
Giulia Rotunno
Cody Rounds
Uldis Rubins
Rolf Saager
Tina Saeidi
Yoshifumi Saijo
Inga Saknite
Kayvan Samimi
Danuta Sampson
Rowan Sanderson
Angelo Sassaroli
Krishnan Sathiyamoorthy
Travis Sawyer
Ilyas Saytashev
Alexander Schill
Hinnerk Schulz-Hildebrandt
Augustino Scorzo
Eric Seibel
Sourya Sengupta
Babak Shadgan
Ruibo Shang
Anna Sharikova
Vladislav Shcheslavskiy
Shuhao Shen
Jindou Shi
Mengjie Shi
Paul Shin
Marina Shirmanova
Manmohan Singh
Sunil Singhal
Koneswaran Sivagurunathan
Thomas Smart
Christopher Smith
Jason Smith
Zachary Smith
Anthony Song
Janet Sorrells
Janis Spigulis
Ronald Sroka
Samuel Streeter
Aleh Sudakou
Chia-Wei Sun
Jialue Sun
Kung-Bin Sung
Kidan Tadesse
Qinggong Tang
Martin Thunemann
Chao Tian
Kenneth Tichauer
Alessandro Torricelli
Vassiliy Tsytsarev
Valery Tuchin
Alon Tzroya
Matthew Urban
Yuriy Ushenko
Urs Utzinger
Pieter van der Zaag
Ton Van Leeuwen
Gijs Van Soest
Gracie Vargas
Jenu Varghese Chacko
Paula Villarreal
Martin Villiger
Phuong Vincent
Anitha Vulugundam
Alec Walter
Anna Wang
Jingyi Wang
Shang Wang
Wenbo Wang
Yaning Wang
Zhaoqiang Wang
Zhuoying Wang
Robert Weersink
Rong Wei
William Winfree
Jungeun Won
Chuhua Wu
Xiang Wu
Yicong Wu
Marvin Xavierselvan
Fei Xia
Jun Xia
Yuchen Xiang
Yijing Xie
Guan Xu
He Xu
Minghao Xue
Vladislav Yakovlev
Feng Yan
Bin Yang
Junjie Yao
Xincheng Yao
Abdul-Amir Yassine
Derrick Yong
Jonghee Yoon
Jie Yuan
Nanxue Yuan
Zhen Yuan
Jooyeong Yun
Jason Zara
Haishan Zeng
Jitao Zhang
Limin Zhang
Oumeng Zhang
Yi Zhang
Yide Zhang
Jianhua Zhao
Kehao Zhao
Min Zhao
Mingjun Zhao
Qingliang Zhao
Yanyu Zhao
Tianyi Zheng
Wenhan Zheng
Caigang Zhu
Jiang Zhu
Quing Zhu
Timothy Zhu
Yihua Zhu

